# Cholesteatoma in the Sellar Region Presenting as Hypopituitarism and Diabetes Insipidus

**DOI:** 10.1097/MD.0000000000002938

**Published:** 2016-03-11

**Authors:** Xiangyi Kong, Huanwen Wu, Wenbin Ma, Yongning Li, Bing Xing, Yanguo Kong, Renzhi Wang

**Affiliations:** From the Department of Neurosurgery (XK, WM, YL, BX, YK, RW), and Department of Pathology (HW), Peking Union Medical College Hospital, Chinese Academy of Medical Sciences, Beijing, China.

## Abstract

Clinically significant sellar cysts unrelated to pituitary adenomas are uncommon. Intracranial cholesteatomas are also rare and are most common in the middle ear and mastoid region. We report an even rarer case of cholesteatoma in the sellar region—a challenging diagnosis guided by clinical presentations, radiological signs, and biopsy, aiming at emphasize the importance of considering cholesteatoma when making differential diagnoses of sellar lesions.

We present a case of cholesteatoma in the sellar region in a 56-year-old man with hypopituitarism, diabetes insipidus, and cystic imaging findings. It was difficult to make an accurate diagnosis before surgery. We present detailed analysis of the patient's disease course and review pertinent literature.

The patient underwent a surgical exploration and tumor resection through a transsphenoidal approach. Pathologic results revealed a cholesteatoma. The patient's symptoms improved a lot after surgery, and the postoperative period was uneventful. Taken together, the lesion's imaging appearance, pathological characteristics, and clinical features were all unique features that lead to a diagnosis of cholesteatoma.

As we did not see such reports by Pubmed and EMBASE, we believe this is the first reported case of sellar cholesteatoma presenting in this manner. This article emphasized that cholesteatomas, although rare, should be considered part of the differential diagnosis of sellar lesions.

## INTRODUCTION

In 1828, Cruveilhier reported the first description of “tumeurs perlees.”^1^ Several years later, after recognizing the presence of cholesterol crystals in a diploic epidermoid, Mueller introduced the term “cholesteatoma.”^1^ This term refers to a destructive and expanding growth consisting of keratinizing squamous epithelium, mainly found off midline in the extra-axial subdural regions—most commonly the cerebellopontine angle cistern (40–50%)—followed by the parasellar/middle fossa space (10–15%).^2,3^ Cholesteatomas typically occur between the ages of 20 and 60, with a peak incidence among those in their 40s. There is no predilection for either sex.^1^ Although cholesteatomas are not, strictly speaking, tumors or cancers, they can still cause significant problems because of their erosive and expansile properties. Here, we report a rare case of cholesteatoma originating in the sella turcica with cystic imaging findings that caused severe headache, secondary hypothyroidism, central hypogonadism, and central diabetes insipidus (DI). The lesion was successfully resected by the transsphenoidal route. To the best of our knowledge, this is the first reported case of sellar cholesteatoma presenting in this manner. The unique disease course and magnetic resonance imaging (MRI) characteristics are presented. The epidemiology, origins, and treatment of cholesteatomas are also discussed.

## CASE PRESENTATION

**FIGURE 1:**
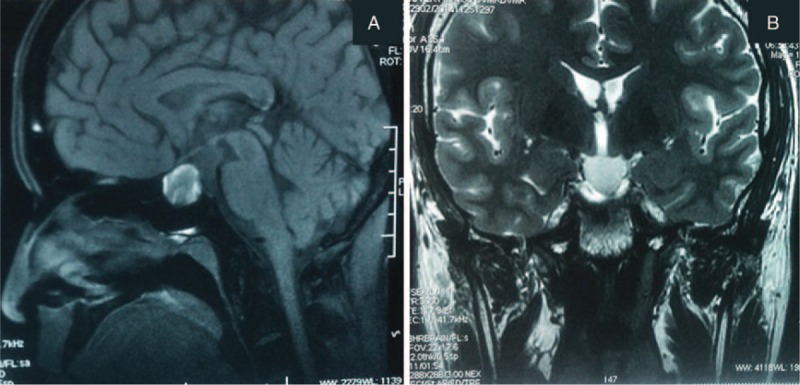
MRI evaluation of the sellar region before surgery. The mass measured 13 mm × 13 mm × 18 mm. The lesion's interior was cystic, which showed as isointense gray matter mixed with hyperintensity on T1-weighted image (A) and showed iso-high T2 signal (B).

A 56-year-old Chinese man, with no notable medical history, had experienced severe headache, fatigue, and poor mental state for about 1 year. The pain was mainly focused in the forehead and could be alleviated by rest or analgesics. He also complained of mildly decreased bilateral visual acuity (VA) in recent months. He said he had not experienced any unconsciousness, convulsions, epilepsy, cognitive disorder, nausea, vomiting, numbness, or constitutional symptoms. On further questioning, he reported a long history of extreme thirst, urinary frequency, and occasional episodes of enuresis that he was too embarrassed to bring to the attention of a health care practitioner. He finally sought medical help at our hospital after 2 weeks of erectile dysfunction. No relevant special circumstances regarding his family history or personal history were identified. Upon ophthalmologic examination, the right VA was 1.0 and the left was 0.6. Mild blepharoptosis and temporal upper quadrant incomplete hemianopia of the left eye and some temporal scotomas of the right eye were observed. Pupillary light reflex and corneal reflex were insensitive on the left side. Neurological examination showed comparatively decreased facial sensation on the left side, especially at the regio zygomatica. Limb movement function and sensory function were normal. Both superficial and deep tendon reflexes were normal. Pathological signs and ataxia were absent. Table 1 shows the results of laboratory tests, including endocrine tests (mean values was measured on 3-time visits to make sure its levels were stable), 24-hour antidiuretic hormone (ADH), complete blood count and blood biochemistry. Free thyroxine (T4) and total T4 were significantly decreased. The sex hormones luteinizing hormone (LH), testosterone (TSTO), dehydroepiandrosterone sulfate (DHEAS), and progesterone (P) were all much lower than the normal, which was consistent with the patient's fatigue and ED. The ADH level was 7.7 μU/24 h (reference range: 11–30 μU/24 h). A mild hypernatremia was noted (152 mmol/L, reference range: 135–145 mmol/L) possibly due to polyuria. Liver and kidney function tests and complete blood count were normal. Thyroid ultrasonography did not reveal any abnormalities. Based on the patient's polyuria, polydipsia, and nocturia, a tentative diagnosis of DI was made. To confirm, 24-hour urine production was measured and increased at 4.7 L per 24 hours (normal ≤3 L per 24 hours). Reduced urine osmolality of 276 mmol/kg (normal >300 mmol/kg) was detected. Then, because the water deprivation test (WDT) is the most reliable method for diagnosing DI, this patient was deprived of water for 8 hours, during which time no significant changes in water loss or urine osmolality were observed. After vasopressin (AVP) administration, the patient showed a reduction in urine output and an increase in urine osmolality of >50%. Therefore, central DI was confirmed. On MRI (Figure 1), T1-weighted images (T1WI) showed a mass in the sphenoid sinus with cephalad extension to the sella turcica, measuring 13 mm × 13 mm × 18 mm and signals of isointensity mixed with hyperintensity. There was a mild mass effect on the optic chiasm, greater on the left than the right. The left cavernous sinus was involved. There was no enhancement of the lesion with administration of gadolinium. Meanwhile, T1WI showed reduced signal in the posterior pituitary; the bright spot due to stored hormones in the neurosecretory granules of normal subjects was absent. The T2-weighted images (T2WI) revealed an iso-high signal mass with a cystic appearance. Differential diagnoses included pituitary macroadenoma, pituitary cyst, cranial buccal cleft cyst, suprasellar cistern hernia, and craniopharyngioma.

**TABLE 1:**
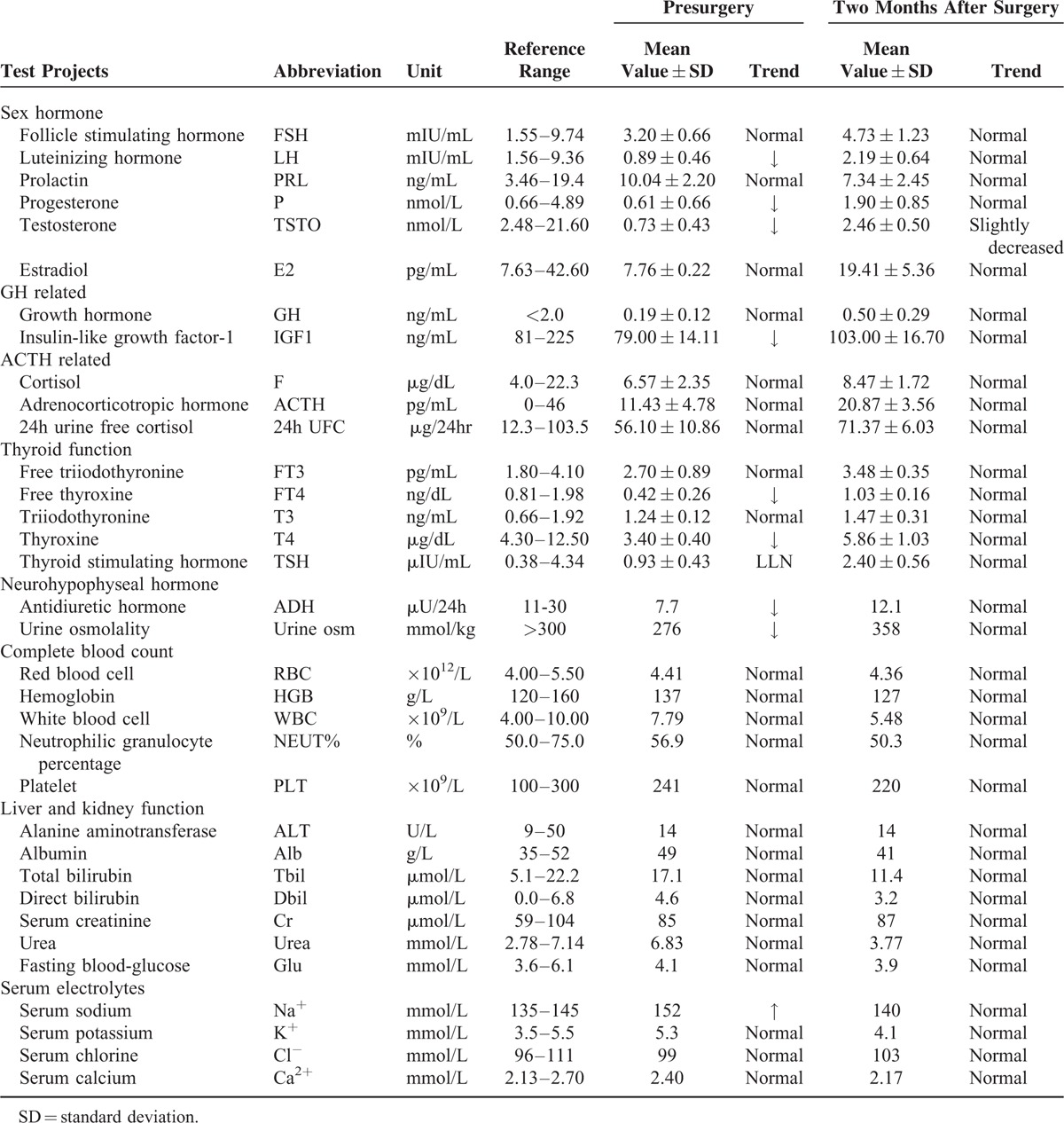
Laboratory Tests Results Before Surgery and 2 Months After Surgery

## CLINICAL COURSE

**FIGURE 2:**
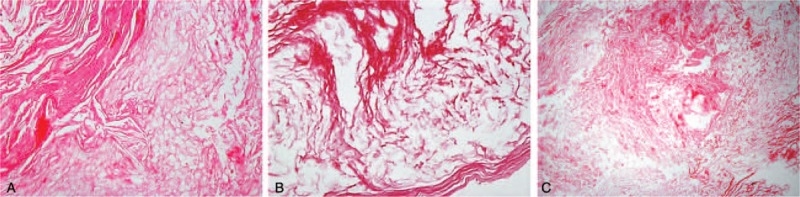
Postoperative paraffin section pathology revealed a combination of keratinous material and stratified squamous epithelium and confirmed the diagnosis of cholesteatoma. H&E staining, ×100 (A and B); ×40 (C).

To relieve the patient's impaired pituitary function, severe headache, and central DI, the patient underwent a surgical exploration and resection. The sublabial transsphenoidal approach was adopted. Upon entering the spheroid sinus, yellow adherent cheesy material was encountered. No hemorrhage or cystic fluid was found. The floor of the sella was eroded and the dura was visible. Under direct fluoroscopy, the anterior, posterior, and superior extent of the lesion was curetted. No cerebrospinal fluid was observed. The cavity was filled with a free graft and the sellar floor was reconstructed with a piece of nasal septal cartilage. The specimen was composed of dry, flaky, cheesy materials, and soft tissue's tiny fragments. Postoperative paraffin section pathology revealed a combination of keratinous material and stratified squamous epithelium and confirmed the diagnosis of cholesteatoma (also referred to as epidermoid cyst, Figure 2). The patient's urine volume gradually decreased to 1.5 to 2 L/24 hours and his headaches resolved completely. The patient reported no side effects of surgery and was dismissed from the hospital 7 days later in good clinical condition. The postoperative period was uneventful. Pituitary functions had almost returned to normal at the 2-month follow-up (Table 1, mean values of 3-time measurements for 3 consecutive days).

## DISCUSSION

Although Cruveilhier described cholesteatoma as a “pearly” tumor in 1828, a cholesteatoma is not a neoplasm, nor does it contain any cholesterol. Cholesteatoma is defined as a cyst-like mass or benign tumor lined with stratified squamous epithelium, usually keratinizing, and filled with desquamating debris.^4^ Although it can remain clinically silent until quite advanced, a cholesteatoma eventually destroys neighboring structures, giving rise to a number of complications, some of which can be life-threatening. Intracranial cholesteatomas are rare, representing 0.21% of primary neoplasms of the central nervous system, and are most common in the middle ear and mastoid region, secondary to trauma or infection that heals improperly and results in invaginated epithelium.^3,5^ Cholesteatomas with sellar extension and acute neurological deterioration caused by such a slow growing mass are less common. This clinical presentation warrants the addition of this lesion to the differential diagnosis of a patient who presents with a sellar mass and symptoms suggestive of pituitary disease.

Intracranial cholesteatomas are generally thought to result from the inclusion of ectodermal elements at the time of closure of the neural groove, or later during the formation of the cerebral vesicles.^6^ These cysts may result from iatrogenic or traumatic implantation of epidermis into the subarachnoid space, mainly along the median neuroaxis.^6^ Most cholesteatomas, however, are located more paramedian, and this is hypothesized to be due to the transplantation of epidermoid cell nests laterally by the migrating otic vesicles or the vasculature. Cholesteatomas in the sellar region are usually attributable to the proliferation of foci of squamous cell rests in the anterior hypophysis. Some of these rests present with cornification and keratohyalin typical of epidermoid tumors and metaplasia of anterior hypophyseal cell is very likely to be the source.^7^

Typically, cholesteatoma symptoms are initially mild. Clinical symptoms are generally caused by the local mass effect of the lesion on adjacent brain tissue or intracranial neurovascular elements and are likely to present once the lesion has reached an appreciable size.^8^ Extension of a cholesteatoma to the optic chiasm may result in visual deterioration or visual field defects. As the internal carotid artery (carotid siphon), and cranial nerves III, IV, V (branches V1 and V2), and VI all pass through the cavernous sinus, its involvement or compression may cause cranial nerve findings like oculomotor paralysis.^9^ Compression on the pituitary gland from the cholesteatoma can cause gland failure, leading to pituitary insufficiency (hypopituitarism).^10^ The symptoms depend on which hormone is involved.^10,11^ Generally speaking, growth hormone (GH) is the first affected pituitary hormone in hypopituitarism patients, causing growth disorders in children and leads to abnormal body composition in adults. The following disorder is usually gonadotropin deficiency, leading to menstrual disorders and infertility in women and decreased sexual function, infertility, and loss of secondary sexual characteristics in men, as in our case. Thyroid stimulating hormone (TSH) and adrenocorticotropic hormone (ACTH) deficiency usually develop later in the course of hypopituitarism. TSH deficiency can lead to hypothyroidism, and thus appetite loss, weight gain, fatigue, and decreased mental function. In our case, the thyroid function tests showed low total T4 and free T4 and the TSH was at the low level of the normal range. Like our case, when lesions involve the posterior pituitary, polyuria, and polydipsia reflect loss of vasopressin secretion. Additionally, a sellar cholesteatoma can cause symptoms of increased intracranial pressure, causing various types of headache.

On computed tomography (CT), a typical sellar cholesteatoma may exhibit a homogeneous low density focus with a smooth, well-defined boundary. Although MRI features were not typical in the present case, they are considered to provide the superior soft tissue differentiation necessary to distinguish between intracranial suppurative complications and cholesteatomas.^12^ Cholesteatomas have a variety of signal intensities (around 50% high, 25 low, the other 25% show an equal T2 signal intensity).^12–14^ Due to the slow growth, the peripheral edema zone is usually not evident. Ten percent to 25% of cholesteatomas cases are calcified.^14^

The ideal treatment of choice for cholesteatomas is surgical excision on the basis of the MRI features.^15^ The microscopic transsphenoidal approach is highly recommended, because it allows for total or subtotal tumor removal under a clear field of vision, and is relatively simpler and easier than craniotomy when the lesion invaded the lateral cavernous sinus without high morbidity.^16^ A chemical meningitis may occur if the tumor cyst fluid overflows to the subarachnoid space.^17^ During the perioperation, corticosteroids are considered to be an effective prophylactic therapy. In spite of operation, cholesteatoma recurrence incidence is still high, in some reports up to 71%.^18^ Many neurosurgeons consequently recommend to perform a “second exploration” 6 to 18 months later for resection of residual tumor tissue.^19^

## CONCLUSIONS

Although uncommon, cholesteatoma should be considered part of the differential diagnosis of sphenoid sinus or sellar region masses. Clinical symptoms are generally due to the local mass effect of the tumor, and hypopituitarism and DI are also common manifestations. Cholesteatoma has variable signal intensity on MRI, and pathological results remain the “gold standard.” We recommend the microscopic transsphenoidal approach for removal of epidermoids of the sellar region.
